# Model development to assess the impact of a preventive treatment with sarolaner and moxidectin on *Dirofilaria immitis* infection dynamics in dogs

**DOI:** 10.1186/s13071-025-06734-x

**Published:** 2025-03-12

**Authors:** Emilie Hendrickx, Thomas Geurden, Cedric Marsboom

**Affiliations:** 1 Aviagis, Risschotlei 33, 2980 Zoersel, Belgium; 2 Zoetis, Mercuriusstraat 20, 1930 Zaventem, Belgium

**Keywords:** Heartworm, Dog, Mosquito, Model, Transmission, Disease, Sarolaner, Moxidectin

## Abstract

**Background:**

*Dirofilaria immitis* is a mosquito-transmitted filarial parasite causing heartworm disease in dogs. The parasite may cause a significant disease burden to the dog population in high prevalence areas and is mainly managed through prophylactic treatments.

**Methods:**

In this modelling study, the effect of a prophylactic treatment with moxidectin and sarolaner on heartworm disease dynamics was investigated in dogs. A compartmental model was developed to investigate different epidemiological settings considering different values for prevalence and host preference.

**Results:**

When the mosquito host preference to dogs is low, a treatment compliance of only 40% decreases the proportion of infectious dogs. When the host preference of the mosquitoes however increases, an exponential increase in infectious dogs was observed, and a higher treatment compliance is required. In high transmission environments, with a high prevalence and a high mosquito host preference, a high treatment compliance (up to 100%) is required to have an impact on the number of infected animals. Notably, in scenarios with higher host preference towards dogs, more mosquitoes are exposed to sarolaner through the blood meal, leading to higher mortality of these mosquitoes and resulting in fewer infected and infectious dogs.

**Conclusions:**

The preventive efficacy, as measured by the number of non-infected dogs, increases with increasing treatment compliance, but the extent of the treatment effect differs with the epidemiological setting. Adding sarolaner to a heartworm prevention has a complimentary impact on mosquito survival and heartworm disease transmission, although this effect depends on the epidemiological settings, emphasizing the true complexity of disease dynamics of a vector-borne disease.

**Graphical abstract:**

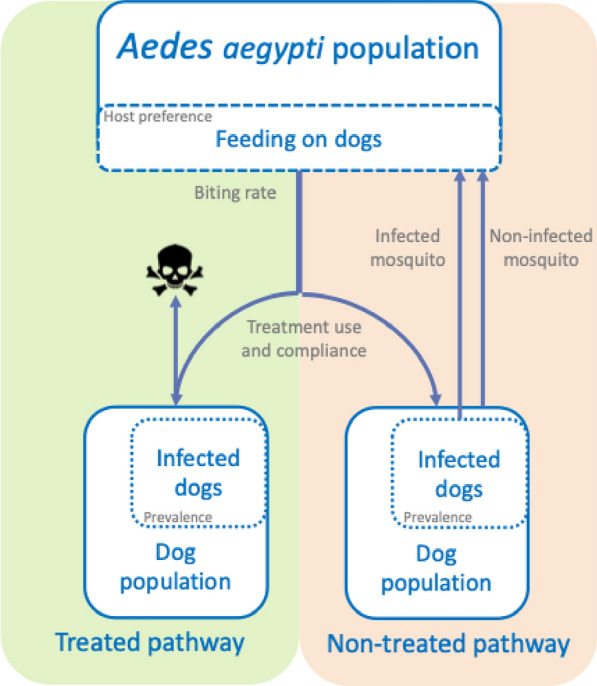

**Supplementary Information:**

The online version contains supplementary material available at 10.1186/s13071-025-06734-x.

## Background

Heartworm disease in dogs is a vector-borne disease caused by *Dirofilaria immitis* and may result in coughing, difficult breathing, exercise intolerance, pulmonary hypertension, right-sided congestive heart failure and mortality [1, 2]. Up to 60 mosquito species have been described as potential vectors for heartworm disease, with *Aedes* spp., *Anopheles* spp. and *Culex* spp. as the most relevant vectors in the USA [3–5]. Due to the high mean temperature and humidity as well as the presence of suitable vectors [6, 7], heartworm infection in the USA is most prevalent around the Gulf Coast and in Southern states [8].

Considering the severity of the disease and the risk of life-threatening complications during treatment of adult heartworm, prevention of infection is preferred, which can be achieved through a direct effect on larval heartworm stages or an effect on the vector. A direct effect on the larval heartworm stages is best achieved through moxidectin because of its efficacy against susceptible as well as resistant heartworm isolates [9–12]. Isoxazolines are systemic ectoparasiticides with known efficacy against fleas, ticks and mites [13]. Recent studies demonstrated their lethal effect against mosquitoes [14–17]. Furthermore, treatment with sarolaner ensures high efficacy against *Aedes* spp. mosquitoes within 72 h after the blood meal for at least 28 days, enabling the prevention of oviposition [14]. Similar effects on egg-laying have been published for fluralaner [18]. Consequently, an isoxazoline treatment may have an impact on local mosquito populations and subsequently on heartworm infections [14, 16], including transmission of resistant heartworm isolates. Similarly, the decrease of malaria infection in the human population through the control of vectors has been described [19].

In the current study, a compartmental model was developed to assess the potential impact of a prophylactic treatment consisting of a combination of moxidectin and sarolaner on heartworm disease dynamics in dogs in different epidemiological settings. Initial insights were generated on the possible complimentary effect of sarolaner on the mosquito populations feeding on treated dogs.

## Methods

### Model development

#### Development of compartmental model

**Fig. 1:**
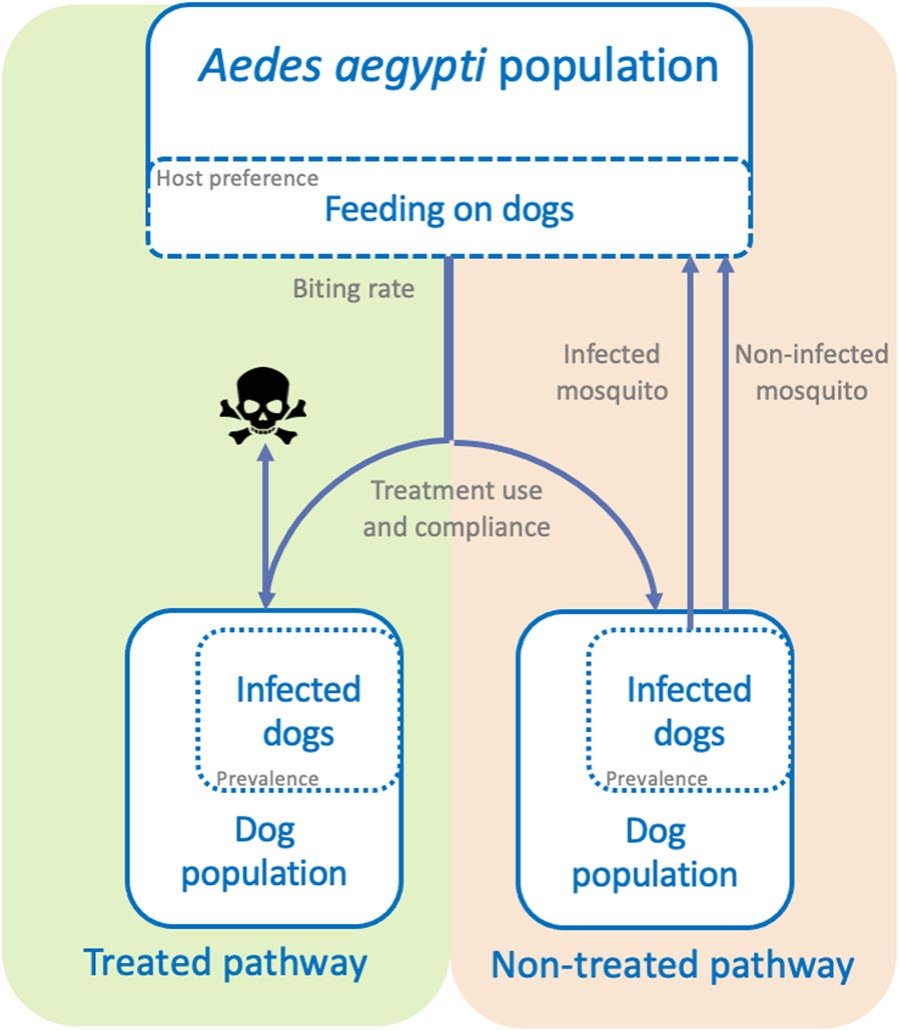
Compartmental transmission model with a treated (moxidectin/sarolaner) and not-treated pathway

A compartmental model (Fig. 1) was developed comprising two transmission pathways.

In each transmission pathway, a proportion (defined by the host preference) of the *Aedes aegypti* mosquito population is expected to feed on dogs, of which a proportion is infected with *D. immitis*. When a mosquito feeds on a non-infected dog, the mosquito remains uninfected. When a mosquito feeds on an infected dog, the mosquito may become infected. In the non-treated pathway, there is no medical interference with the heartworm transmission cycle, and the mosquito will be able to complete its gonotrophic cycle. In the treated transmission pathway, all or a proportion of the dogs receive a combination of moxidectin and sarolaner. It is expected that sarolaner will be 100% effective against *Ae*. *aegypti* mosquitoes for at least 28 days and that mortality will occur within 72 h [14]. Since sarolaner has no mosquito-repellent properties, the mosquito will be able to take in a full blood meal and potentially infect the dog with heartworm. Moxidectin has 100% efficacy against larval heartworm stages and therefore can prevent the establishment of a new infection in treated dogs [2, 9, 12]. While data on fluralaner and prevention of egg laying [18] are available, no combination product with efficacy against heartworm is currently available. No data on prevention of mosquito egg prevention are available for other isoxazolines. Therefore, the model focussed on a combination of sarolaner and moxidectin (Simparica Trio®).

Heartworm has a 6–9-month pre-patent period in dogs, indicating that dogs may be infected but not yet able to transmit infection during that period of time (not infectious). In the current model, infected dogs are in the pre-patent period and infectious dogs are infected with patent infections. Nevertheless, it was assumed that all non-treated dogs acquiring an infection would become infectious, and the effect of the prophylactic treatment was evaluated based on a change in the proportion of infectious dogs. Similarly, after ingesting the infected blood meal, the mosquito becomes infected, but it takes another 6 days for the mosquito to become infectious. In both the dogs and mosquitoes, an infected and infectious compartment in the respective populations was considered in the model. Mosquitoes were divided into four different categories: non-infected, infected, infectious and treated mosquitoes (who will die within 72 h) and four different age classes, which all have a maximum lifespan of 24 days. The model also included five dog categories, i.e. non-infected dogs (treated and untreated), infected dogs (treated and untreated) and infectious dogs (untreated), able to transmit the infection to mosquitoes. Age differences were not accounted for in dogs, although it was considered that dogs must be at least 6 months to become infectious. Each model etended over 61 time steps, with each time step encompassing 6 days. While it is acknowledged that the introduction of infected mosquitoes into an environment cannot be avoided [20, 21], this model considered an isolated mosquito population. The same applies to dog migration; consequently, it is possible that the modelling scenarios will evolve to an end stage where all dogs end up either infectious or remaining uninfected. This must be considered when interpreting the modelling results.

#### Epidemiological parameters considered in the compartmental model

After the development of the compartmental model, a list of epidemiological parameters was defined. Parameter values were obtained through a literature review in PubMed and Google Scholar and through expert opinion. An overview of the parameters considered in the model is provided in Table 1, of which three were considered as a variable throughout the model assessment. The host preference (HP) is a parameter measuring the feeding preference of the mosquitoes to different hosts.

**Table 1:** Parameters used in the compartmental model

Parameter	Values	Sources
*Aedes aegypti* parameters		
Minimum duration for infected mosquito to become infectious	6 days	[30]
Longevity *A. aegypti*	< 24 days	[31]
Host preference	0.25 VAR (0.02–0.50)	[5, 22–24]
Biting rate	0.83	[32]
Minimal infectious rate	0.32	[33]
Birth rate	0.35	Expert’s opinion
Death rate (natural death)	0.1	Expert’s opinion
Mosquito death rate after sarolaner ingestion	100% within 72 h	[14, 16]
Dog-related parameters		
*Dirofilaria immitis* prevalence	0.1 VAR (0.08–0.34)	[1, 25–27]
Minimal infectious rate in dogs	1	[34]
Treatment compliance	0.6 VAR (0.1–0.9)	[27]

A higher HP in dogs leads to a higher disease transmission rate in an endemic environment. Several *Ae. aegypti* HP studies have been published [5, 22–24], and the HP to dogs is reported to vary from 2 to 50%. This parameter has a high uncertainty in the model. The second parameter was the *D. immitis* prevalence (DP) in dogs in the Mississippi Delta (Southern US states including Georgia, Alabama, Louisiana, Mississippi, Tennessee, Arkansas), which is reported to vary from 8% in veterinary clinics [1, 25] to 34% in animal shelters [26, 27]. Concerning treatment compliance (TC), 60% of the dog owners provided prophylactic treatment for their pets at the veterinary clinic but only 40% complied with a treatment schedule as recommended by the American Heartworm Society [27].

#### Model sensitivity analysis

The sensitivity of the model for HP and DP was analysed while maintaining TC at a constant rate of 40%. During this sensitivity analysis, the impact of 5% incremental changes of one parameter on the shape of the curves was assessed while maintaining the other parameter at the median value described in the literature. The HP was modelled from 5 to 40% while the DP parameter was set to 21%. The DP was also modelled from 5 to 40%, while the HP was set to 25%. After this sensitivity analysis with 5% increments, incremental steps of 2% were performed for certain intervals. Finally, the sensitivity of the model to a combination of parameters was assessed. The aim of this sensitivity analysis was to assess the impact of these model parameters on the modelling results.

### Modelling the impact of treatment compliance in four different epidemiological scenarios

In this study, four epidemiological scenarios were considered (as outlined in Table 2) to assess the impact of treatment compliance on the disease dynamics in dogs and the potential impact on mosquito populations.

**Table 2:** The four epidemiological scenarios modelled with different host preferences and *Dirofilaria immitis* prevalence

	Host preference (%)	*Dirofilaria immitis* prevalence (%)
Scenario 1	25	8
Scenario 2	14	8
Scenario 3	2	8
Scenario 4	25	34

Each scenario had a unique combination of DP and mosquito HP, which were kept constant while varying the treatment compliance from 40 to 80% with 5% incremental steps. In the first three scenarios (Table 2), an 8% heartworm DP as observed in veterinary practices in the Mississippi Delta area [1, 25] was replicated. Across the range described in the literature, three HP values were included: 25% (scenario 1; mean of the combined literature data—see Table 1); 14% (scenario 2) and 2% (scenario 3) as the lowest HP value [28]. A fourth scenario was developed in which a 34% heartworm DP in shelters in the Mississippi Delta [26, 27] was replicated. As previously suggested [24], when the dog-human ratio is high, mosquito feeding on dogs increases. Therefore, a high value for HP was used in scenario 4. Maximum value for HP according to the literature was 50%, but when considering the high uncertainty for this parameter and the fact that increasing the HP above 25% only resulted in marginal changes of the modelling results, an HP of 25% was used in scenario 4.

As 100% compliance in a dog population is not readily achieved, the effects of different prophylactic treatment compliance ratios on the potential transmission of heartworm were evaluated in these four epidemiological scenarios by comparing the proportion of infectious dogs to the proportion of the not infected dogs. Ideally, the proportion of not infected dogs is maximised as opposed to the proportion of infectious dogs, i.e. a minimum number of dogs will be newly infected and maximum number of dogs remain uninfected. First, the impact of increasing the treatment compliance from 40 to 60% was evaluated. According to the literature, 60% of the dog owners purchase the prophylactic treatment but only 40% correctly and compliantly administer the treatment they purchase. The impact of further increasing the treatment compliance was evaluated in the different scenarios.

## Results

### Model sensitivity analysis

The model is most sensitive to lower HP values. Increasing the HP with 5% increments resulted in significant changes in the shape of the curves until an HP of 20%. Further increasing the HP had less impact on the modelling outputs. The same applies for heartworm DP, i.e. higher sensitivity for lower DP values, decreasing sensitivity with increasing DP values. This was further confirmed by performing an additional sensitivity analysis for both parameters at their lower values with 2% incremental steps. Combining both parameters increases the model’s sensitivity significantly compared to their isolated attribution to the model’s output. A more elaborate description of the sensitivity analysis and corresponding modelling results can be found in the supplementary information.

### Modelling the impact of treatment compliance in four different epidemiological scenarios

**Fig. 2:**
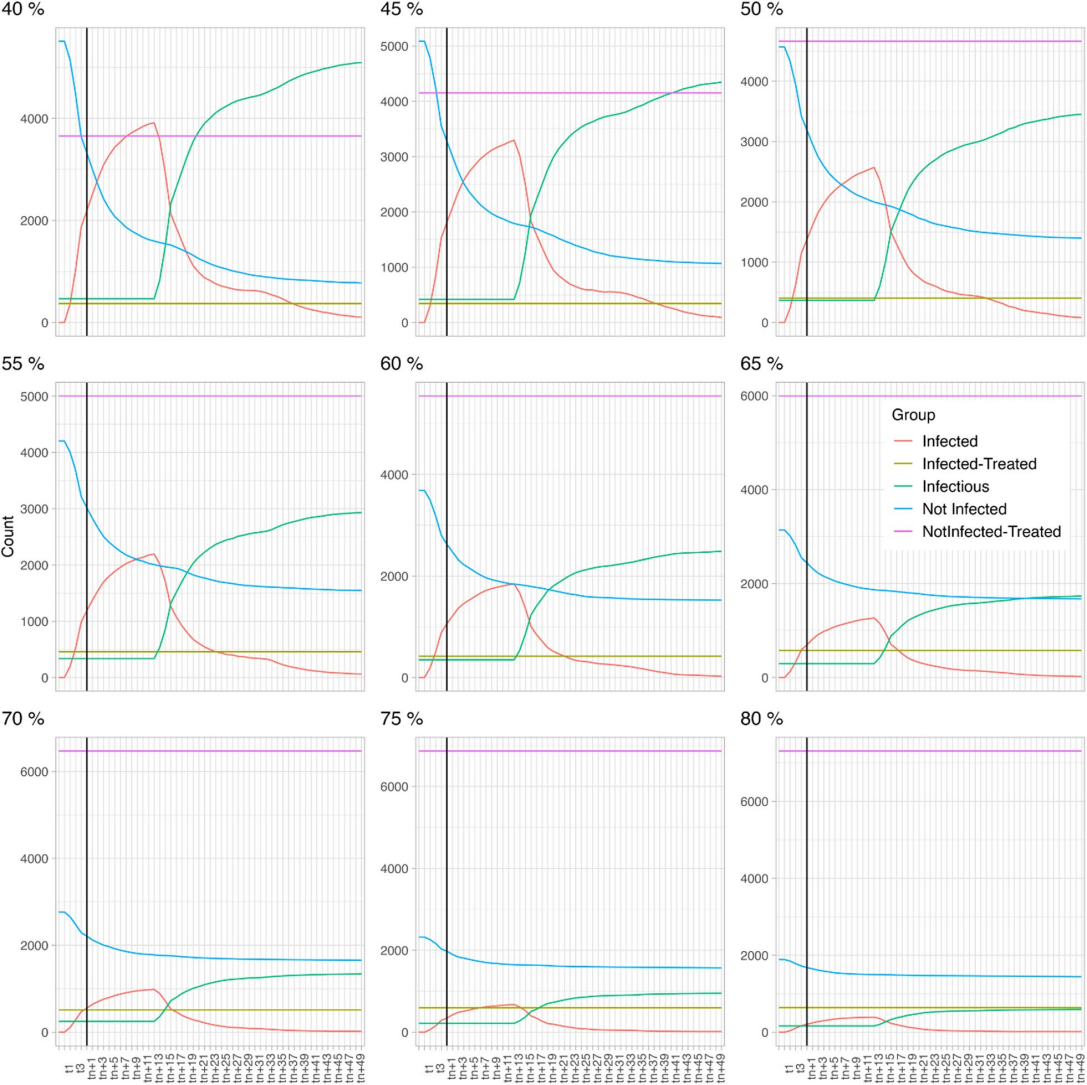
Scenario 1 (host preference fixed at 25% and disease prevalence at 8%) with varying treatment compliance (40–80%). Each model extended over 61 time steps (X-axis), with each time step encompassing 6 days

In scenario 1 (HP 25% and DP 8%, see also Fig. 2), at a TC of 40%, the number of infectious dogs (green line) is significantly higher than the proportion of non-infected dogs (blue line). If the TC is increased to 60%, the proportion of infectious dogs is still higher than that of the non-infected dogs but the difference between both groups of dogs decreases significantly. A minimum treatment compliance of 70% is required to ensure that the proportion of infectious dogs is lower than the proportion of non-infected dogs. Increasing to an 80% treatment compliance has additional benefits as it further decreases the proportion of infectious dogs.

**Fig. 3:**
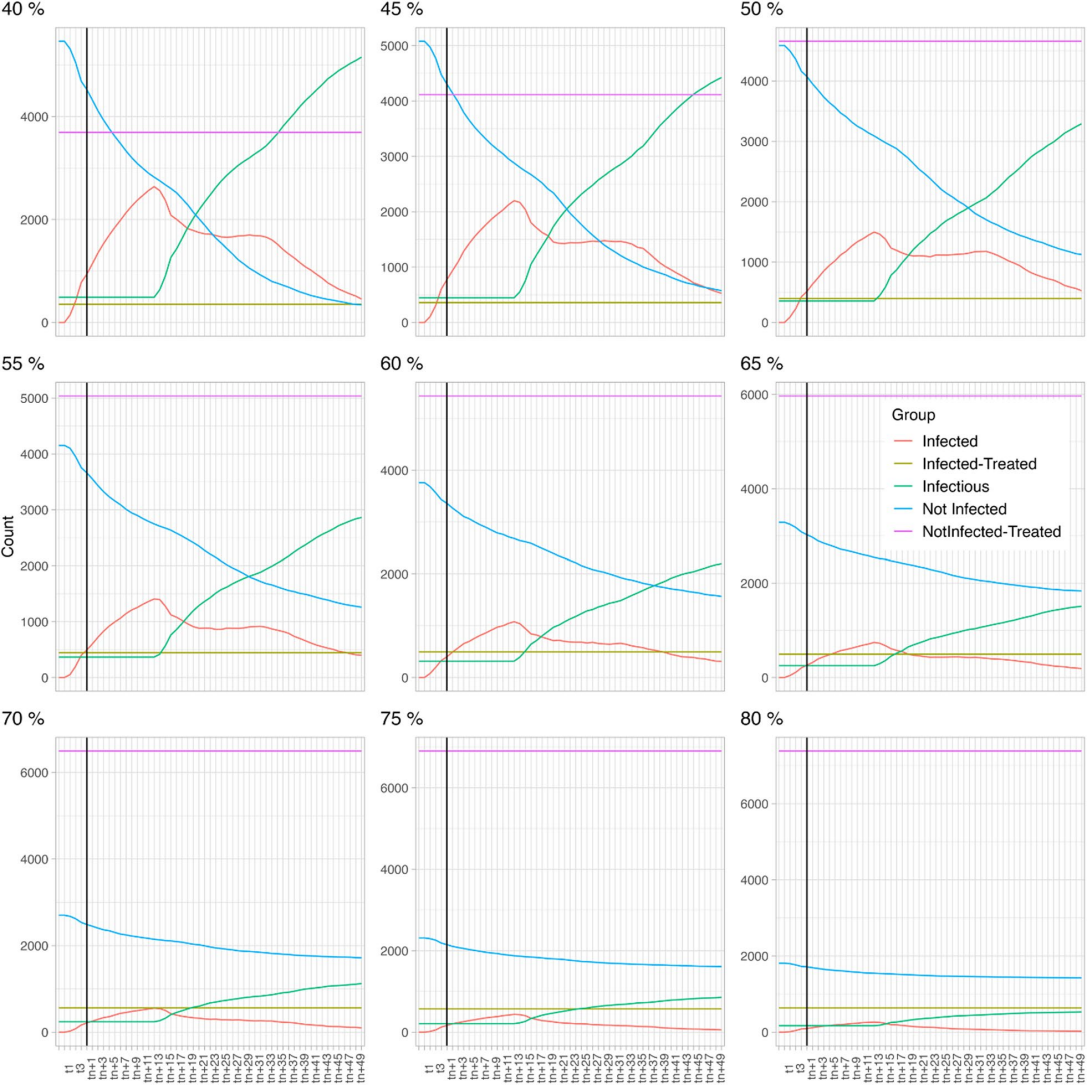
Scenario 2 (host preference fixed at 14% and disease prevalence at 8%) with varying treatment compliance (40–80%). Each model extended over 61 time steps (X-axis), with each time step encompassing 6 days

In scenario 2 (HP is 14% and DP is 8%; see also Fig. 3), at a 40% TC, the proportion of infectious dogs (green line) is significantly higher than the proportion of non-infected dogs (blue line). Notably, when this is compared to the 40% TC of the first scenario, the total number of infected dogs is higher in this second scenario, despite the lower HP. If the TC is increased to 60%, a similar dynamic as in scenario 1 is observed, i.e. the proportion of infectious dogs is still higher than for the non-infected dogs, but the difference decreases. A minimum TC 70% is required to decrease the proportion of infectious dogs below that of the not infected dogs and increasing the TC to 80% further decreases the proportion of infectious dogs.

**Fig. 4:**
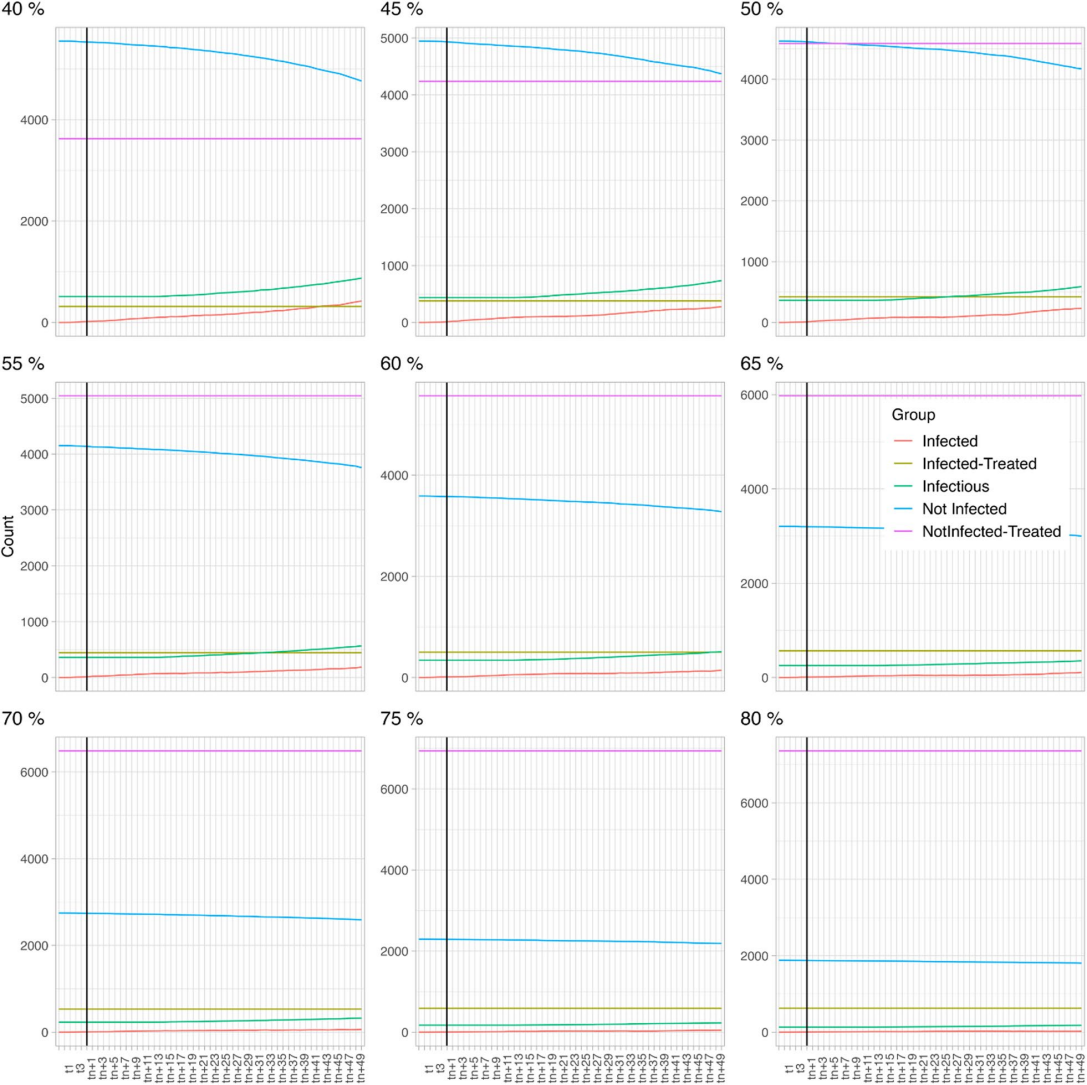
Scenario 3 (host preference fixed at 2% and disease prevalence at 8%) with varying treatment compliance (40–80%). Each model extended over 61 time steps (X-axis), with each time step encompassing 6 days

In scenario 3 (HP is 2% and DP is 8%, see also Fig. 4), 40% treatment compliance results in a significantly higher number of not-infected dogs than infectious dogs, whereas in scenarios 1 and 2 this was only achieved at 70% treatment compliance. The curve does not reach an equilibrium within the time frame of this modelling study; consequently, we cannot confidently state that once the equilibrium is reached the proportion of non-infected dogs will still be higher than the proportion of infectious dogs. The same applies for increasing treatment compliance. The curves flatten with increasing treatment compliance, meaning that the numbers of infectious dogs increase more slowly.

**Fig. 5:**
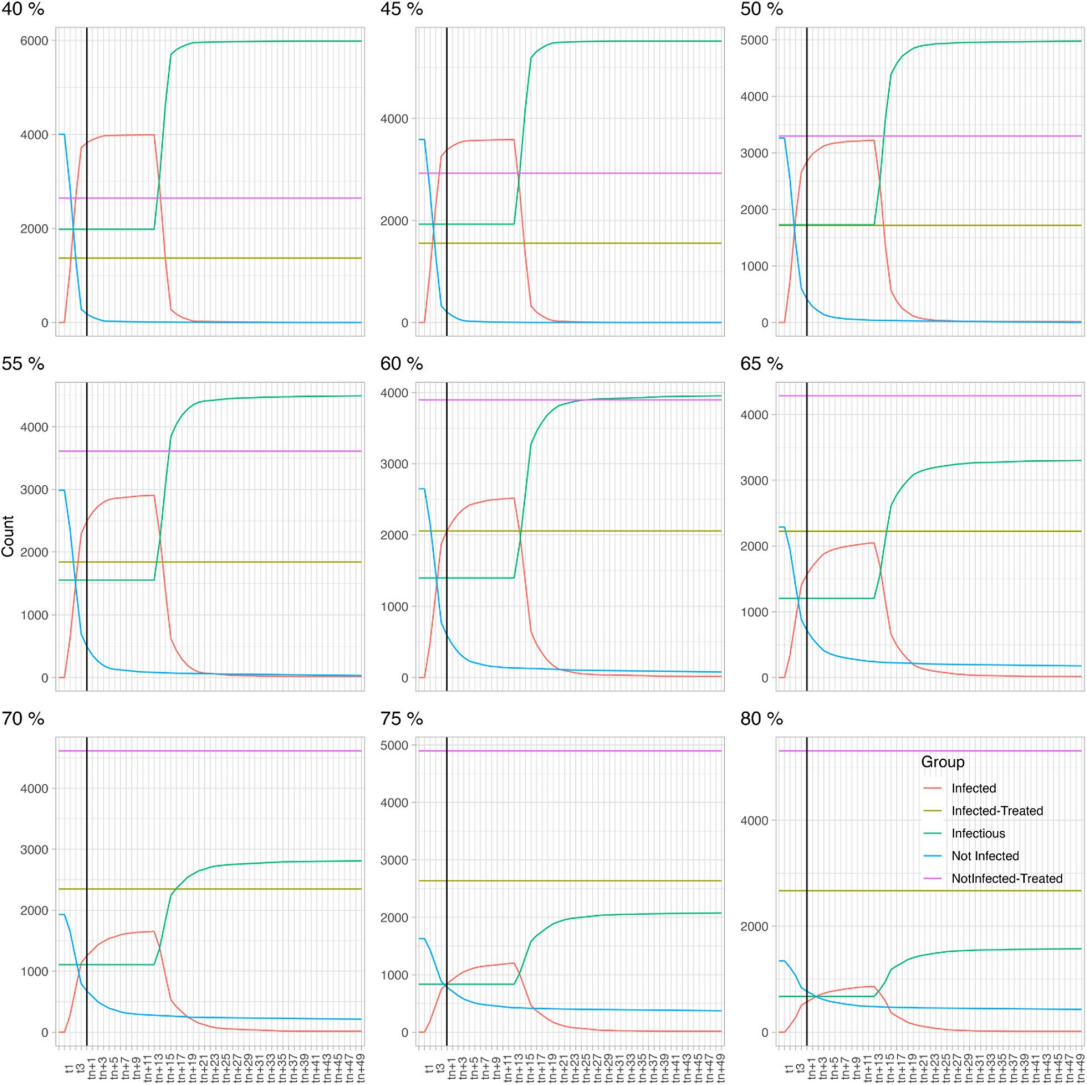
Scenario 4 (host preference fixed at 25% and disease prevalence at 34%) with varying treatment compliance (40–80%). Each model extended over 61 time steps (X-axis), with each time step encompassing 6 days

In scenario 4 (HP is 25 and heartworm DP is 34%, see also Fig. 5), the opposite is observed; at a TC of 40% all dogs become infectious (green line) quickly and no dogs remain non-infected (blue line). Increasing to a TC of 60% only slightly postpones the moment at which all dogs become infectious. The impact of further increasing the TC is obvious as a proportion of the dogs indeed remains non-infected. However, even at a TC of 80% the proportion of infectious dogs remains higher than the proportion of non-infected dogs. If considered as an isolated population in this study, i.e. not including mosquito and dog migratory data, maximising the TC to 100% would result in a completely healthy population.

**Fig. 6:**
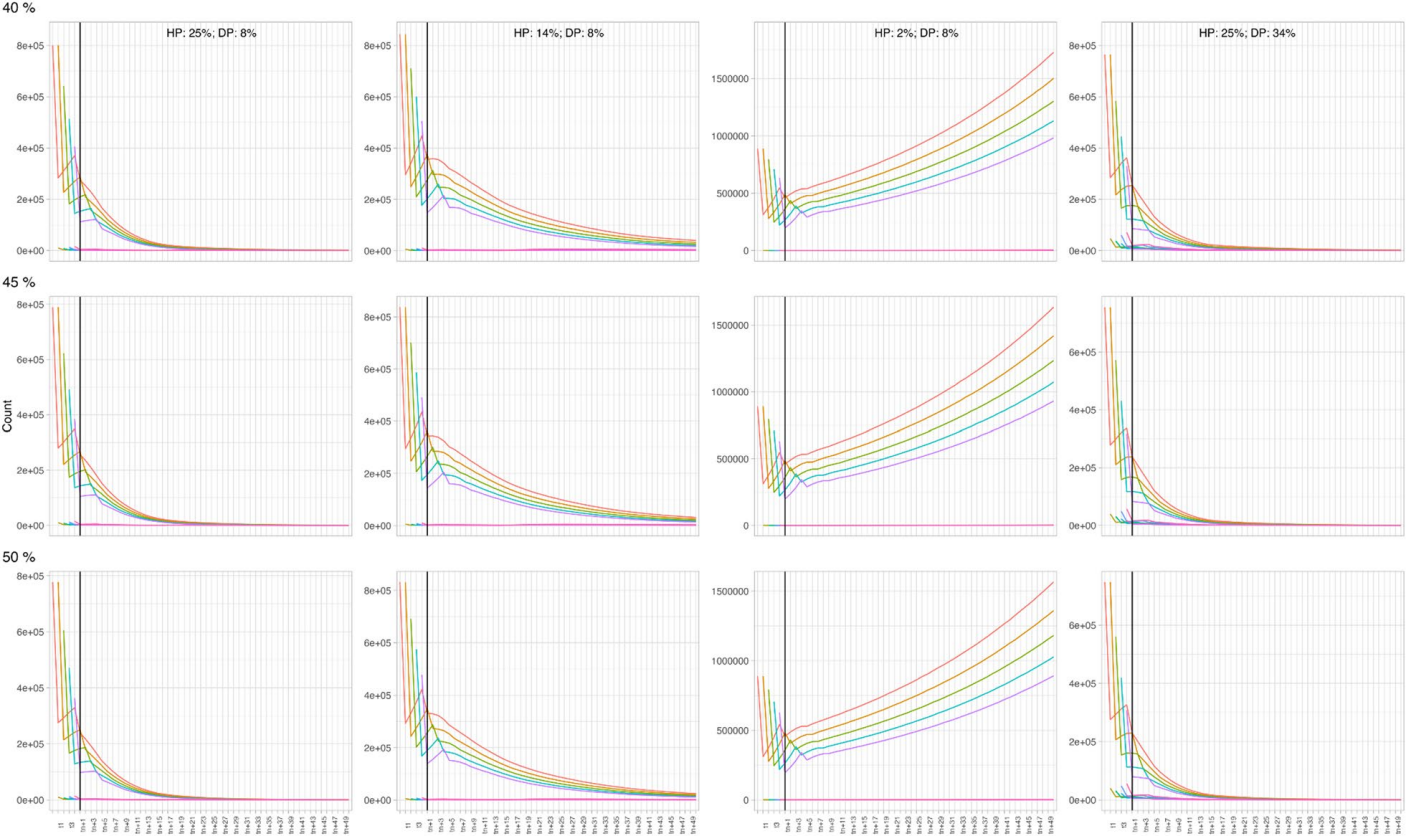
The evolution of the mosquito population in the four different scenarios for treatment compliance of 40–50%. Each model extended over 61 time steps (X-axis), with each time step encompassing 6 days

The mosquito population dynamics were plotted for each of the scenarios (Fig. 6). Only the results for TCs of 40%, 45% and 50% are provided as further increasing TC did not result in significant changes.

The different lines on each graph represent the different mosquito age groups. The initial deviation of the curves is part of the model initialisation period before it stabilises. This is a normal phenomenon in modelling and results are only interpreted past this phase. In scenario 1 (first column), the mosquito population dropped quickly. In the second scenario, a similar trend was observed, but slower. In scenario 3 the mosquito population increased over time. Finally, in scenario 4 the mosquito population decreased quickly. Within each of these scenarios, the TC had no significant impact on the mosquito population dynamic.

## Discussion

In this study a compartmental model with two transmission pathways was developed considering the impact of TC, mosquito HP and DP on heartworm disease dynamics in dogs. The limitations of this compartmental model are acknowledged and include the absence of data on mosquito and dog movements in an endemic area. Therefore, some of the modelling scenarios evolve to an end-stage plateau phase, whereas fluctuations over time through new introductions of dogs or mosquitoes are to be expected in the field. Furthermore, more detailed data on mosquito HP would improve the accuracy of the modelling output as this is currently the parameter with the largest confidence intervals. Finally, only the *Ae. aegypti* mosquito population was considered in the model, as currently the efficacy of sarolaner has only been evaluated against this mosquito species [14].

Despite its limitations, the compartmental model does enable an assessment of the relative impact of varying TC ratios considering four epidemiological scenarios with different mosquito HP and DP values. In scenarios 1 to 3, a constant DP of 8% was used. It was observed that if the mosquito HP to dogs is low (2%—scenario 3), a TC of 40% in the dog population resulted in a decrease of the proportion of infectious dogs. When the mosquito HP however increased to 14% (scenario 2) or 25% (scenario 1), the number of infectious dogs increased, and a treatment compliance of only 40% resulted in a significant proportion of the dog population becoming infectious. In these HP scenarios (14% and 25%), increasing the TC to 60% significantly reduced the proportion of infectious dogs, although the proportion of infectious dogs remained higher than for the non-infected group. Higher TC (> 70%) further decreased the proportion of infectious dogs. These results confirm that in highly endemic areas, TC is indeed a key parameter for successful prevention of heartworm disease.

In scenario 4, a high DP scenario (34%) was considered as well as a high transmission environment, such as shelters in an endemic area. As the number of dogs is higher compared to the number of alternative hosts, mosquitoes will feed proportionally more on dogs [24, 26, 27, 29], resulting in a high mosquito HP (25%). In this scenario, increasing the TC from 40 to 60% and even beyond 75% had a minor impact on the proportion of infectious and non-infected dogs, with the proportion of infectious dogs remaining significantly higher than for the non-infected dogs. Compared to scenarios 1 to 3, a high TC (up to 100%) is expected to be needed in this high transmission environment. Without a migration component of dogs and mosquitoes, a high TC would however bias the modelling outcome towards an uninfected dog population. Therefore, a 100% TC was not modelled as only isolated dogs and mosquito populations were considered in this model.

The prophylactic treatment aims to lower the proportion of infected dogs mainly by the effect of moxidectin on immature heartworm stages, but a complimentary impact on the disease dynamics through the lethal effect of sarolaner on mosquitoes has been observed in this study. When comparing the total number of infectious dogs in scenario 1 and 2 at equal TC (e.g. 40%), more dogs become infectious in scenario 2 even though the HP is half of that in scenario 1. Because of the higher HP in scenario 1, one would expect to have more infected animals. However, it does make sense that if the HP towards dogs is higher, more mosquitoes will be exposed to sarolaner through the blood meal leading to mortality of these mosquitoes [14], resulting in a fewer infected and infectious dogs. In these specific conditions, sarolaner has a complimentary impact on the transmission of heartworm, including resistant isolates. The direct impact of sarolaner on the mosquito population was also observed in scenario 4. However, in this epidemiological scenario, the decrease of the mosquito population did not result in a decrease of the number of infected dogs, probably because of the significantly higher DP. This emphasises the true complexity of disease dynamics of a vector-borne disease. These findings suggests that the prophylactic treatment with sarolaner has a complementary impact on mosquito population and on the disease transmission dynamics, depending on the epidemiological settings.

## Conclusions

The modelling results show the impact of a combined treatment with moxidectin and sarolaner on the DP in dogs. As expected, there was an impact of increasing treatment compliance, albeit the benefit differs between epidemiological settings. Whilst complex mosquito dynamics were not modelled in this study, the results suggested that in certain epidemiological settings sarolaner had an additional benefit on the mosquito population dynamics and on the control of heartworm disease. More detailed data on the effect of sarolaner or other isoxazolines on other mosquito species that transmit heartworm and data on mosquito and dog movement in endemic areas can be used in the future to refine the model.

## Supplementary Information

Supplementary Material 1.

## Data Availability

Data used in the modelling are listed in Table 1. The R code of the model can be requested by contacting the corresponding author.
