# Supplementary material for: Model development to assess the impact of a preventive treatment with sarolaner and moxidectin on Dirofilaria immitis infection dynamics in dogs
Source: Parasit Vectors. 2025 Mar 12;18:102. doi: 10.1186/s13071-025-06734-x (PMC11900526; doi:10.1186/s13071-025-06734-x)
Supplement: Supplementary file 1 — Supplementary Material 1. [file 13071_2025_6734_MOESM1_ESM.docx]

**Supplementary Information**

**Additional file S1: Sensitivity analysis of the compartmental model**

1. Mosquito Host Preference (HP) sensitivity analysis (Figure A)

The model was most sensitive to lower HP values. When comparing the model output from HP 5% to HP 15% the shape of the curves significantly changed. When the HP is 5% both the infectious and not infected curves do not evolve to a plateau phase within the time frame of the modelling study, whereas this is the case for a HP of 15%. When comparing the curves of the higher HP values (>15%) less variability in the modelling outputs was observed. Every scenario evolved to a plateau phase for both the infectious and not infected groups. And the level at which these plateaus settle was the same for every curve, specifically the number of infectious animals maximises and none of the animals remain uninfected. With each incremental step the plateau phase was reached faster but with decreasing difference.

**Figure A:** Mosquito host preference sensitivity analysis with a Disease Prevalence fixed at 21% and a Treatment Compliance fixed at 40%.

**
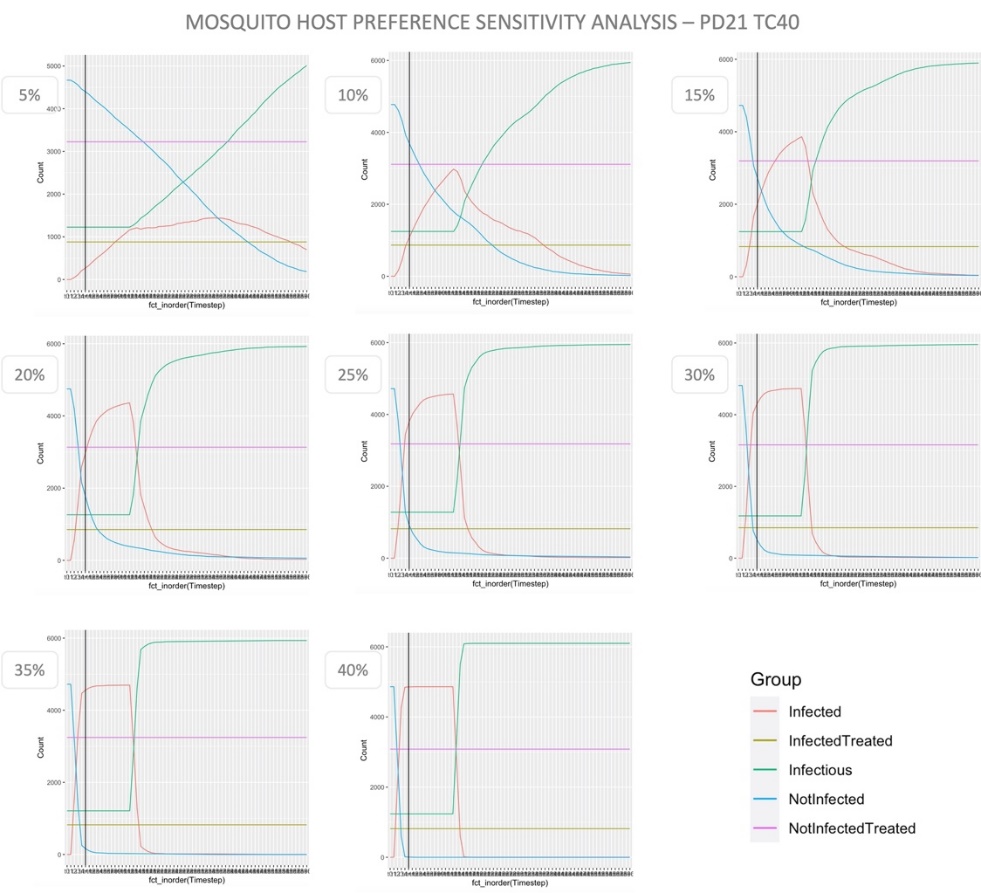
**

1. Disease Prevalence (DP) sensitivity analysis (Figure B)

The sensitivity of the model to DP in dogs was comparable to that of the HP parameter. The plateau phases for infectious and not infected dogs was reached earlier compared to HP, at a DP of 10%. But the level of the plateau only maximises (maximal infectious and minimal not infected (i.e. 0)) at a DP level of 20%. When comparing the curves of the higher DP values (>20%) less variability in the modelling outputs was observed. Every scenario evolved to a plateau phase for both the infectious and not infected groups. The level at which these plateaus settled was the same for every curve, specifically the number of infectious animals maximised and none of the animals remained not infected.

**Figure B:** Disease Prevalence in dogs sensitivity analysis with Host Preference fixed at 25% and Treatment Compliance at 40%

**
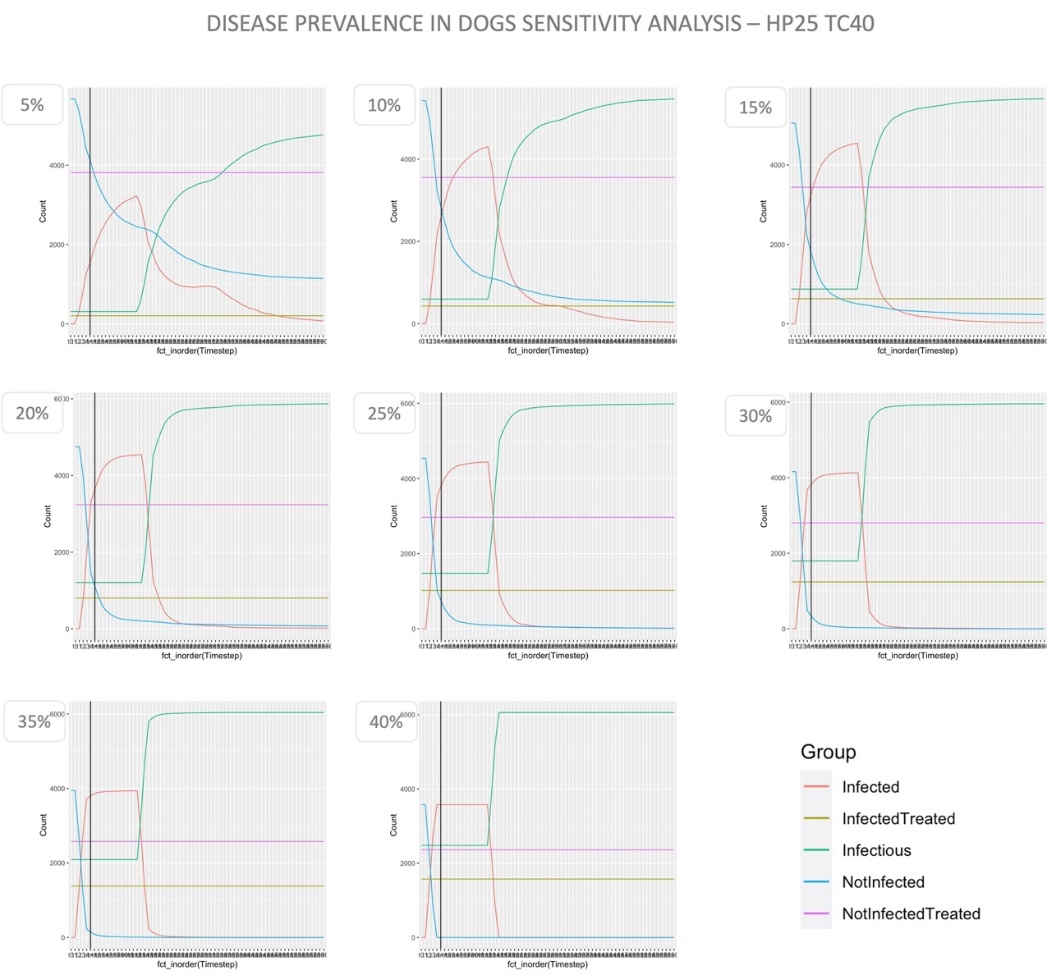
**

1. HP and DP sensitivity in lower values (Figure C)

The HP and DP parameters in the model’s sensitivity was highest for the lower values, an additional set of models was run. The sensitivity of the model for the lower values of HP and DP was further compared for the values of 5, 7 and 9% for both parameters. Based on the changes in the shape of the curves no significant difference in model sensitivity was observed. The fact that the model was more sensitive to lower HP and DP values was confirmed.

**Figure C:** Comparing model sensitivity for Host Preference and Disease Prevalence at a Treatment Compliance of 40%


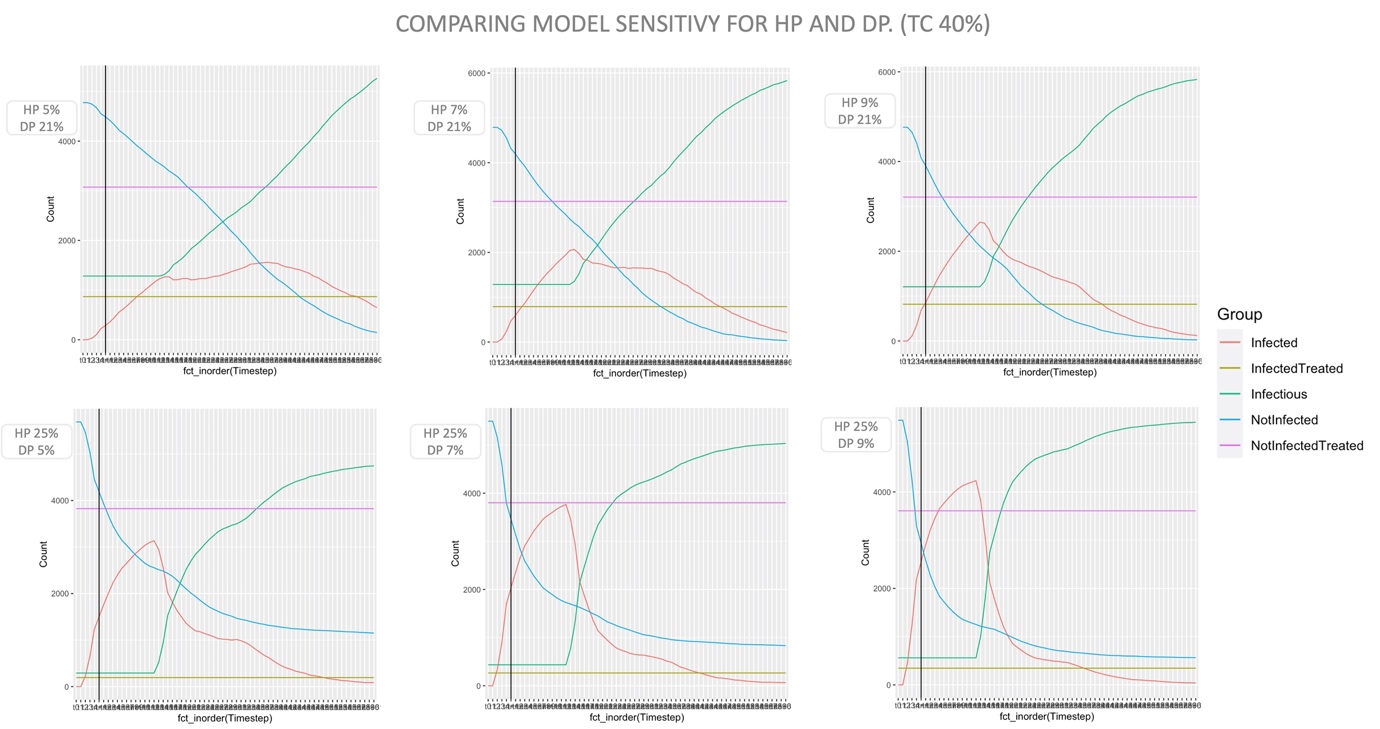


1. HP and DP combined sensitivity analysis (Figure D)

In the second part of the sensitivity analysis, the combined effect of HP and DP on the model output was assessed, with TC fixed at 40%. For each parameter four cut-off values were determined (2, 18, 34 and 50%), with 2% being the lowest HP value, 34 and 50% being the highest DP and HP values, respectively. 18% was chosen to get proportional incremental steps. When observing the modelling output, it become clear that the combination of both parameters increased the sensitivity of the model’s output significantly, as compared to their isolated attribution in the model’s output (Fig A and B). Based on these first model runs, the model’s sensitivity for HP and DP was comparable, but the combination of both parameters clearly has a significant impact on the model output. Therefore, the importance of the combination of these parameters needs to be considered as they don’t occur in a vacuum. These parameters interact with each other in a significant way, small changes in a combination of parameters might still have a significant impact on the overall result.

**Figure D:** Combining both Host Preference and Disease Prevalence to evaluate model sensitivity.

**
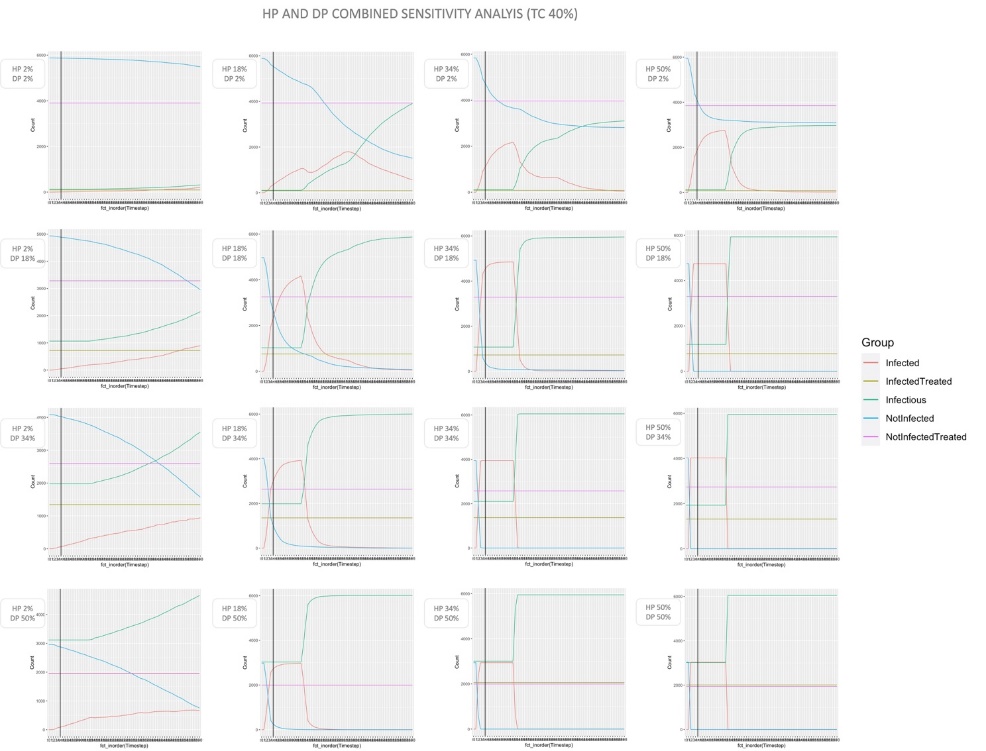
**
